# Isoprostanes as Biomarker for Patent Ductus Arteriosus in Preterm Infants

**DOI:** 10.3389/fped.2020.00555

**Published:** 2020-09-08

**Authors:** Caterina Coviello, Maria Luisa Tataranno, Iuri Corsini, Valentina Leonardi, Mariangela Longini, Francesco Bazzini, Giuseppe Buonocore, Carlo Dani

**Affiliations:** ^1^Division of Neonatology, Careggi University Hospital of Florence, Florence, Italy; ^2^Department of Neonatology, University Medical Center Utrecht, Utrecht, Netherlands; ^3^Department of Pediatrics, Obstetrics and Reproductive Medicine, University of Siena, Siena, Italy; ^4^Department of Neurosciences, Psychology, Drug Research and Child Health, Careggi University Hospital of Florence, Florence, Italy

**Keywords:** isoprostanes, patent ductus arteriosus, oxidative stress, ibuprofen, preterm infants

## Abstract

**Context:** It has been reported that isoprostanes (IPs) have a role in the pathophysiology of ductus arteriosus during the fetal and neonatal period. Our aim in this study was to assess if urinary IPs (uIPs) levels correlate with the risk of developing a hemodynamically significant patent ductus arteriosus (hsPDA) in preterm infants.

**Materials and methods:** Infants with 23 + 0 – 33 + 6 weeks of gestational age and respiratory distress syndrome (RDS) were consecutively enrolled. Urine samples were collected on the 2nd and 10th day of life (DOL) for uIPs measurement. Echocardiography for hsPDA diagnosis was performed between 24 and 48 h of life. Regression analysis was performed to assess the correlation between uIPs and hsPDA. Receiver operating characteristic (ROC) curve analysis was used to evaluate the accuracy of the uIPs in predicting the occurrence of hsPDA.

**Results:** Sixty patients were studied: 33 (55%) developed a hsPDA, 27 (45%) had ibuprofen hsPDA closure, and six (10%) required surgical closure. uIPs levels decreased from the 2nd to the 10th DOL. Adjusted regression analysis demonstrated that uIPs on the 2nd DOL were associated (*p* = 0.02) with the risk of developing a hsPDA. A cut-off level of 1627 ng/mg of creatinine of uIPs predicted the development of a hsPDA with a sensitivity of 82% and a specificity of 73%.

**Conclusion:** Early measurement of uIPs on the 2nd DOL is a reliable biomarker of hsPDA development and, alone or combined with other markers, might represent a non-invasive tool useful for planning the management of PDA in preterm infants.

## Introduction

Patent ductus arteriosus (PDA) is a frequent complication in preterm infants with respiratory distress syndrome (RDS) and 60–70% of preterm infants of <28 weeks' gestation receives medical and/or surgical treatment for PDA (1). The proper management of PDA is the subject of lively debate because randomized controlled trials (RCTs) of PDA closure using non-steroidal anti-inflammatory drugs (NSAIDs) have often failed to demonstrate relevant benefits in preterm infants (2). However, a persistent left-to-right shunt through the ductus complicating RDS has been associated with a worsening of respiratory failure, lowering of survival rate, and increased risk of intraventricular hemorrhage (IVH) and bronchopulmonary dysplasia (BPD) (1, 3–6). Therefore, pharmacological closure of PDA is indicated before significant left-to-right shunting occurs.

Currently, functional echocardiography is the best tool to diagnose PDA in preterm infants, to monitor its progress, and to determine its treatment (7). In fact, biomarkers, such as brain-type natriuretic peptide (BNP) and N-terminal pro-BNP (NTpBNP), have a significant range concentration overlap between neonates with no PDA, or small, moderate, and large PDA which limits their clinical application (8).

Recently, it was reported that isoprostanes (IsoPs) have a role in DA closure. IsoPs derive from the free radical-mediated peroxidation of phospholipid-bound arachidonate and, although they are mainly known as a biomarker of oxidative stress, they might induce physiological effects during the fetal and neonatal period, such as DA vascular constriction mediated by stimulation of thromboxane receptors (9). Van der Sterren et al. studied the vasoactive effects of IsoPs in chicken embryo isolated DA and demonstrated that they can induce a strong DA constriction (9). These results were confirmed by Chen et al. who found that oxygen exposure increases IsoPs levels in newborn mouse lung which induce constriction of the isolated term DA through the activation of thromboxane A2 (TxA2) receptor (10). Moreover, Longini et al. found that urinary IsoPs (uIPs) levels decreased after PDA closure with ibuprofen in preterm infants (11).

On the basis of these considerations we hypothesized that uIPs level might predict the development of a hemodynamically significant PDA (hsPDA). Thus, our aim was to assess uIPs levels in a cohort of very preterm infants and correlate them with the risk of hsPDA development.

## Materials and Methods

### Population and Sample Collection

A prospective center-based study was carried out from June 2014 to December 2014 at the neonatal intensive care unit of Careggi University Hospital of Florence. The study was approved by the local ethics committee. Infants with gestational age from 23 + 0 to 33 + 6 weeks with respiratory distress syndrome (RDS) were consecutively enrolled after written parental informed consent. Exclusion criteria were major congenital malformations, chromosomal disorders, inborn errors of metabolism, and acute renal failure.

Urine samples of 2 ml were collected from each infant on the 2nd and 10th day of life (DOL) by placing a gauze in the infants' diaper. The samples were frozen and maintained at −80°C until analysis, which was performed at the Laboratory of Oxidative Stress of the University of Siena, Italy. Urinary isoprostanes (uIPs) were measured using tandem mass spectrometry (GC-MS) according to the methodology described by Casetta et al. (12). Urinary creatinine levels were measured at the two time points using a spectrophotometer method (Creatinine Diacron Kit®, Diacron International, Grosseto, Italy). Isoprostane values were corrected for intersubject differences in renal function and were expressed as nanograms of isoprostanes/mg of urinary creatinine.

### Management of PDA

All enrolled infants underwent echocardiography between 24 and 48 h of life. The diagnosis of hsPDA requiring pharmacological treatment was made by echocardiographic demonstration of a ductal left-to-right shunt, with a left atrium to aortic root ratio >1.3 or a ductal size >1.5 mm (13), excluding the cases in which the closing flow pattern suggested a restrictive PDA (14). All ultrasound studies were performed by pediatric cardiologists or by neonatologists trained in neonatal echocardiography, and the treatment was decided by the neonatologist on duty. Infants with hsPDA received intravenous ibuprofen (Pedea® Orphan Europe, Puteaux, France) at a dose of 10 mg/kg followed by 5 mg/kg after 24 and 48 h. Pharmacological treatment could be repeated at the same dosage. Infants with hsPDA were considered for surgical ligation after failure of the medical therapy.

### General Data Recording

The following clinical and demographic data were also collected by reviewing patients' medical records: GA, birth weight, birth weight <10° percentile, gender, mode of delivery, Apgar score at 5 min of life, fraction of inspired oxygen (FiO_2_) on 2nd and 10th DOL, occurrence and duration of non-invasive respiratory support [nasal continuous positive pressure (NCPAP), bilevel positive airway pressure (BiPAP), nasal intermittent ventilation (NIV)], and mechanical ventilation [patient-triggered ventilation (PTV), high frequency oscillatory ventilation (HFOV)], sepsis, BPD, necrotizing enterocolitis (NEC), intraventricular hemorrhage (IVH), periventricular leukomalacia (PVL), retinopathy of prematurity, and stay-in-hospital duration. Sepsis was defined as positive blood culture. BPD was defined as oxygen requirement at 36 weeks of PMA (15). NEC was defined as Bell's stage >2 (16). IVH was classified according to Papile et al. (17), and PVL according to de Vries et al. (18).

### Statistical Analysis

Patients' characteristics were described as mean and standard deviation (SD), rate and percentage, or median and interquartile range (IQR). The Student's *t*-test or Mann–Whitney *U* test were used to compare continuous data, while *X*^2^ test was used to compare categorical data. Median values, including changes in uIPs levels between 2nd and 10th DOL and between infants with and without hsPDA, were compared using Kruskal–Wallis test. A *p* < 0.05 was considered statistically significant.

Univariate regression analyses were performed to assess the correlation between uIPs and PDA. The following variables were entered into the model: GA, BPD, NEC, IVH, mechanical ventilation, and mean FiO_2_ at samples collection. In the final multivariable regression model, relevant perinatal and neonatal factors that showed an association with uIPs with alpha level <0.05 were included. Data analysis was performed using IBM SPSS Statistics version 20 (SPSS INC, Chicago, Illinois, USA).

To analyze the impact of the ‘uIPs level on the 2nd DOL on the occurrence of hsPDA, we used ROC (receiver operating characteristic) curve analysis. The test's ability to classify patients as those who will develop hsPDA or not is represented by the area under the ROC curve (AUC). The AUC can be in the range of 0.5–1.0, wherein an AUC of 0.5 indicates that the classification model is of no value, and a value of 1.0 indicates perfect diagnostic accuracy. In turn, the cut-off point of the ROC curve indicates the uIPs level that gives the most true and the least false indications of hsPDA development, and therefore has the best predictive power.

*Post hoc* analyses demonstrated that the population size of our study allows an 85% statistical power to detect as significant (*p* < 0.05) the difference between uIPs levels measured on the 2nd and the 10th DOL.

## Results

**Figure 1:**
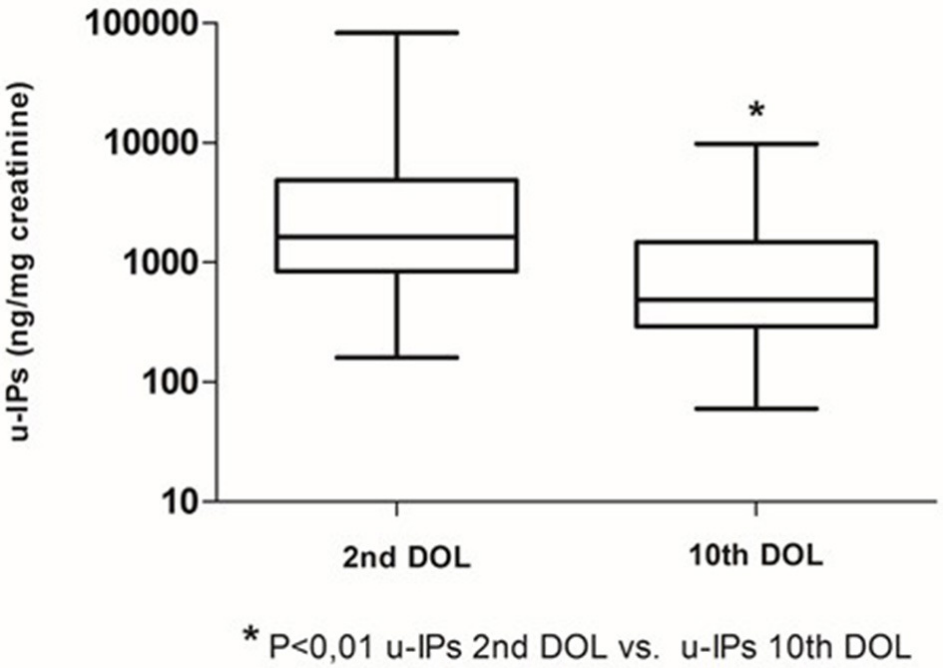
Urinary levels of isoprostanes (uIPs) on the 2nd and 10th DOL. Median values and IQR.

Clinical characteristics of enrolled infants (*n* = 60) are summarized in Table 1. We observed a decrease of uIPs levels from the 2nd to the 10th DOL [1627.2 (842.0–4882) vs. 486.1 (292.8–1467.8)] ng/mg of creatinine (*p* < 0.01) (Figure 1). Significant changes of uIPs from the 2nd to the 10th DOL were also found in the sub-groups of infants who had ibuprofen closure of hsPDA (Table 2).

**Table 1:** Clinical characteristics of the study population.

	**No PDA** **(*n* = 27)**	**hsPDA** **(*n* = 33)**	***P***
Gestational age (weeks)	31 ± 2	27 ± 2	0.007
Birth weight (gr) <10th percentile	1645 ± 335 0 (0)	878 ± 368 7 (21)	0.949
Male	15 (57)	16 (48)	0.301
Cesarean section	19 (73)	22 (66)	0.062
Apgar score at 5 min	9 (8–9)	8 (6–9)	0.004
FiO_2_ on 2nd DOL on 10th DOL	0.24 ± 0.09 0.21 ± 0.02	0.27 ± 0.06 0.23 ± 0.03	0.943 0.063
Mechanical ventilation Duration (day)	3 (11) 0.3 ± 1.4	20 (60) 9.7 ± 22.0	<0.001 0.003
Non-invasive ventilation Duration (day)	20 (76) 4 ± 4	32 (96) 23 ± 22	<0.001 <0.001
Sepsis	2 (7)	11 (33)	<0.001
BPD	0 (0)	11 (33)	<0.001
NEC	0 (0)	3 (9)	0.002
IVH ≥ grade 3	1 (3) 1 (3)	8 (24) 4 (12)	0,005 0.367
PVL	0 (0)	2 (6)	0.002
ROP ≥ grade 3	0 (0) 0 (0)	7 (21) 2 (6)	<0.001 N/A
Mortality	0 (0)	3 (9)	<0.001
Stay in hospital (day)	33 ± 2	72 ± 49	<0.001

*Mean ± SD, rate and (%), or median and (IQR)*.

*GA, gestational age; hsPDA, hemodynamically significant patent ductus arteriosus; BPD, bronchopulmonary dysplasia; NEC, necrotizing enterocolitis; IVH, intraventricular hemorrhage; PVL, periventricular leukomalacia; ROP, retinopathy of prematurity*.

**Table 2:** Urinary levels of isoprostanes (uIPs, ng/mg of creatinine) on the 2nd and 10th DOL in infants without PDA.

	**uIPs on 2nd DOL**	**uIPs on 10th DOL**	***P***
No PDA (*n* = 27)	969.9 (541.0–1470.6)	410.5 (226.0–1027.5)	0.742
Ibuprofen treated PDA (*n* = 27)	2700.0 (1205.7–6688.0)^*^	535.0 (314.0–1791.9)^#^	0.001
Surgical closed PDA (*n* = 6)	5028.7 (1233.0–17770.0) ^**^^∧^	1689.0 (198.5–5541.8)	0.974

*With hsPDA treated with ibuprofen, or who underwent surgical ligation after failure of pharmacological treatment. Median and IQR*.

* *P < 0.01 no PDA vs. ibuprofen treated PDA*;

** *P = 0.01 no PDA vs. surgical closure*.

∧ *P < 0.01 ibuprofen treated PDA vs. surgical closure*.

# *These patients had PDA closed on 10th DOL*.

Fifty-five percentage (*n* = 33) of patients developed a hsPDA, 45% (*n* = 27) had ibuprofen hsPDA closure, and 10% (*n* = 6) failed pharmacological treatment and underwent surgical closure. Eleven patients (18%) received two courses of ibuprofen. Infants with hsPDA had lower GA and Apgar score at 5 min, higher incidence of IVH, PVL, CLD, ROP, NEC, sepsis, mortality, and lengthier stay in NICU compared to infants without PDA (Table 1).

### Isoprostanes and PDA on the 2nd DOL

**Figure 2:**
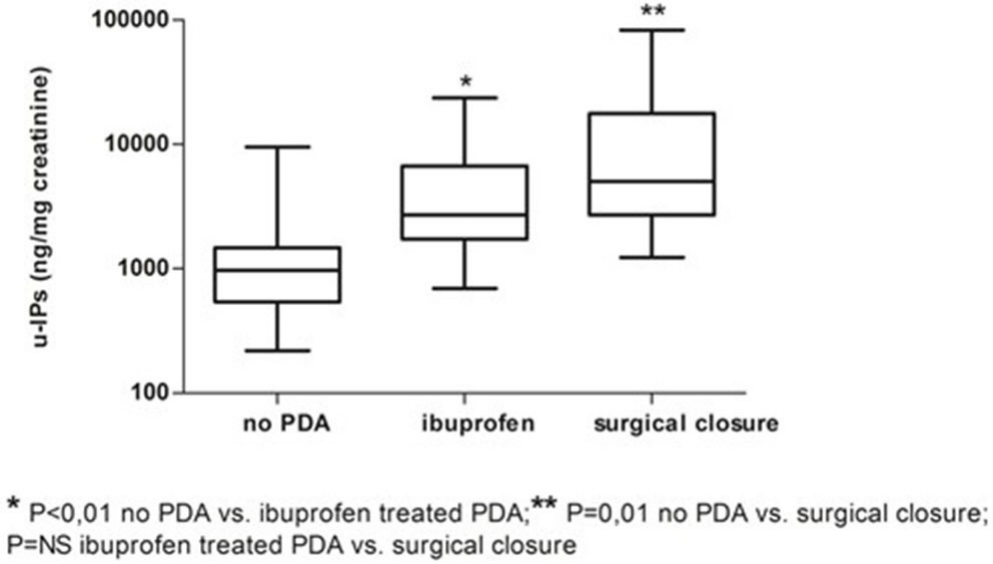
Urinary levels of isoprostanes (uIPs) on the 2nd DOL in infants without PDA, with hsPDA treated with ibuprofen, or who underwent surgical ligation after failure of pharmacological treatment. Median and IQR.

Infants without PDA showed a significantly lower uIPs in comparison with infants with ibuprofen-treated hsPDA and who needed surgical closure (after failure of ibuprofen therapy) (*p* < 0.01) (Table 2 and Figure 2). Univariate regression analyses demonstrated that uIPs sampled on the 2nd DOL were associated (*p* < 0.05) with a lower GA and a higher occurrence of hsPDA. After adjusting for GA, BPD, IVH, NEC, and mean FiO_2_ the association between uIPs and hsPDA remained significant (*r* = 0. 38, B = 2729.8, CI = 416.4–5043,3, *p* = 0.02).

**Figure 3:**
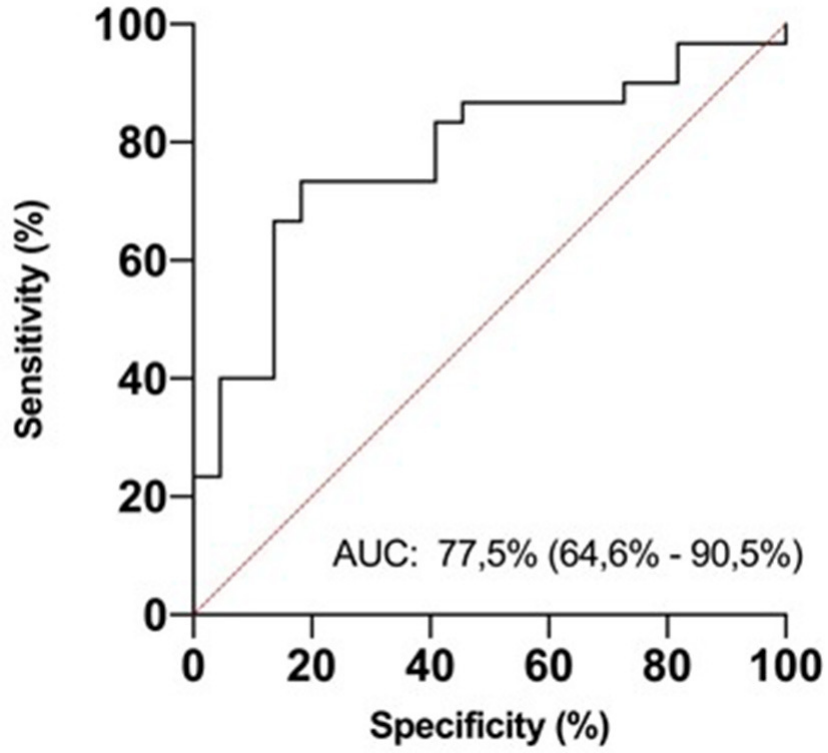
ROC curve analysis for urinary isoprostanes (uIPs) concentration on the 2nd DOL. The area under the curve is 0.775, 95% CI 0.646–0.905. The iPIs levels plotted curve indicated 1,627 ng/mg of creatinine as the best predictive threshold with a sensitivity of 82% and a specificity of 73%. ROC curve discriminates newborns with hsPDA from newborns without it.

In ROC analysis, uIPs in the 2nd DOL significantly predicted hsPDA development, with an AUC of 0.78 and 95% CI 0.65–0.71 (*p* < 0.0001), showing the best prognostic cutoff point at uIPs = 1627 ng/mg of creatinine, with a sensitivity of 82% and a specificity of 73% (Figure 3).

### Isoprostanes and PDA on 10th DOL

Six infants (10%) had hsPDA on the 10th DOL. Urinary IPs were similar in infants with or without hsPDA (Table 2). Univariate regression analyses did not demonstrate any association on the 10th DOL between uIPs and hsPDA. After adjusting for GA, BPD, IVH, NEC, and mean FiO_2_ none of the evaluated variables showed a correlation with uIPs.

## Discussion

This is the first study to investigate the possible predictive role of uIPs levels on the development of hsPDA. We found that uIPs on the 2nd DOL were higher in infants who developed hsPDA than in infants who did not, and were higher in infants who had hsPDA refractory to drug therapy requiring surgical closure than in infants who had pharmacological closure of hsPDA. It is noteworthy that the correlation between uIPs levels on the 2nd DOL and the development of hsPDA remained statistically significant after adjusting for the main potential confounding factors. Thus, uIPs levels might be a valuable biomarker of hsPDA, as confirmed by ROC analysis which demonstrated that a uIPs cutoff level of 1627 ng/mg of creatinine on the 2nd DOL predicts a hsPDA with a sensitivity of 82% and a specificity of 73%.

Isoprostanes are a product of free radical-induced injury by peroxidation of lipids and their synthesis can easily occur in preterm newborns due to their limited antioxidant defenses and large production of free-radicals according to several factors, such as hyperoxia, inflammation, infections, free iron release, and activation of arachidonic acid cascade (19). The role of IsoPs in the DA closure has recently been reported (8, 9), suggesting that they might induce a DA vascular constriction during the fetal and neonatal period. In particular, Chen et al. found that IsoPs can induce constriction of DA through the activation of thromboxane A2 (TxA2) receptor (10). However, they found that IsoPs can also induce vasodilation of the preterm, isolated DA mediated by the prostaglandin E2 receptor 4 (EP4) (10). Hence, as they demonstrated, IsoPs can exert both constrictive and dilatory effects on the DA depending on relative predominance of the TxA2 and EP4 receptors (10). With increasing maturity, the balance between EP4 and TxA2 shifted in favor of the contractile effects of TxA2 stimulation at term gestation. Therefore, it can be speculated that the postnatal transition from the relatively low oxygen intrauterine environment to the significantly higher oxygen extrauterine environment can induce oxidative stress and IsoPs synthesis in newborn infants. This effect could favor DA constriction and closure in term infants via activation of TxA2 receptor or DA dilation and hsPDA development in preterm infants via activation of EP4 receptors.

On the 10th DOL uIPs decreased in comparison with the 2nd DOL levels, and differences between infants without hsPDA, with hsPDA responding to pharmacological therapy, or requiring surgical closure disappeared. This reduction might be due to the maturation of infants who achieved a more favorable balance between anti-oxidant and pro-oxidant factors and overcame the acute phase of respiratory distress syndrome, in agreement with previously reported inverse correlation between IPs synthesis and gestational age (20). Moreover, only in patients who had the pharmacological therapy of hsPDA, the decrease of uIPs level might be due to the antioxidant properties of ibuprofen (11) which is able to scavenge the hydroxyl radical and/or chelates iron (21) and has been found to suppress neuronal oxidative damage more potently than naproxen or acetylsalicylic acid (22).

Our results confirmed the findings of Longini et al. who studied 43 infants with gestational age <33 weeks and hsPDA, and found that uIPs decreased after PDA closure with ibuprofen (11). Moreover, they found that uIPs increased 1 week (from 11.5 to 14 DOL) after the closure and explained this increase with the lack of ibuprofen antioxidant effect after its suspension (11). This latter result is interesting but we cannot comment on it because studies differed in terms of times of urine sampling, drugs used, and occurrence of repeated course of hsPDA pharmacological therapy.

Limitations of our study include the small number infants with hsPDA which precludes the possibility of using regression analysis to evaluate the possible correlation between uIPs levels and the risk of undergoing surgical closure of hsPDA. Moreover, our population has a wide range of gestational ages which might affect the range of uIPs levels. However, we are confident that the high quality of data analyses could contribute to minimize the effects of this limit on our results.

In conclusion, we found that uIPs levels measured on the 2nd DOL are significantly correlated with the risk of developing a hsPDA. We demonstrated that a uIPs cutoff level of 1627 ng/mg of creatinine can predict a hsPDA with a sensitivity of 82% and a specificity of 73%. Early measurement of uIPs, alone or combined with other markers, might represent a reliable, non-invasive biomarker useful for planning the management of PDA in preterm infants.

### Implications for Practice

We found that early and painless measurement of uIPs levels predicts the development of a hsPDA with a sensitivity of 82% and a specificity of 73%. Since the proper management of PDA is continuously debated between neonatologists, the discovery of reliable biomarkers of hsPDA might be useful in the clinical practice in driving its management together with the findings of echocardiography. Thus, if a rapid method for their dosing might be developed, uIPs could be one of these promising biomarkers.

## Data Availability Statement

The raw data supporting the conclusions of this article will be made available by the authors, without undue reservation.

## Ethics Statement

The studies involving human participants were reviewed and approved by Pediatric ethics committee of Tuscany region. Written informed consent to participate in this study was provided by the participants' legal guardian/next of kin.

## Author Contributions

CC, MT, IC, VL, ML, FB, GB, and CD have made substantial contributions to conception and design of the study, critically revised the article, and have given their final approval of the version to be published. CC and CD performed the data statistical analyses and wrote the manuscript. CC, MT, IC, and VL collected data. All authors contributed to the article and approved the submitted version.

## Conflict of Interest

The authors declare that the research was conducted in the absence of any commercial or financial relationships that could be construed as a potential conflict of interest.
